# Interaction of different *Chlamydiae* species with bovine spermatozoa

**DOI:** 10.1186/s12866-019-1392-z

**Published:** 2019-01-25

**Authors:** Thomas Eckert, Sandra Goericke-Pesch, Carsten Heydel, Martin Bergmann, Johannes Kauffold, Klaus Failing, Axel Wehrend

**Affiliations:** 1 Klinikum Veterinärmedizin, Clinic for Obstetrics, Gynecology and Andrology of Large and Small Animals, Justus-Liebig University Giessen, Frankfurter Strasse 106, 35392 Giessen, Germany; 2 Institute for Veterinary-Physiology and Biochemistry, Justus-Liebig University Giessen, Frankfurter Strasse 100, 35392 Giessen, Germany; 3 Department of Veterinary Clinical Sciences, Section for Veterinary Reproduction and Obstetrics, University of Copenhagen, Dyrlægevej 68, 1870 Frederiksberg C, Denmark; 4 Reproductive Unit of the Clinics – Clinic for Small Animals, University of Veterinary Medicine Hannover, Bünteweg 15, 30559 Hannover, Germany; 5 Institute for Hygiene and Infectious Diseases of Animals, Justus-Liebig University Giessen, Frankfurter Strasse 85-89, 35392 Giessen, Germany; 6 Institute for Veterinary Anatomy, Histology and Embryology, Justus-Liebig University Giessen, Frankfurter Strasse 98, 35392 Giessen, Germany; 7 Ambulatorische und Geburtshilfliche Tierklinik, Veterinärmedizinische Fakultät, University of Leipzig, An den Tierkliniken 29, 04103 Leipzig, Germany; 8 Unit for Biomathematics and Data Processing, Justus-Liebig University Giessen, Frankfurter Str., 35392 Giessen, Germany

**Keywords:** *Chlamydiae*, Cattle, Semen motility, CASA (computer assisted sperm analysis)

## Abstract

**Background:**

Interaction of spermatozoa and *Chlamydiae* spp. might contribute to reduced fertility in cattle. To proof this hypothesis, bovine semen was incubated with viable or heat inactivated *Chlamydia* (*C.*) *abortus* or *psittaci* (Multiplicity of infection = 1) and sperm motility was monitored with a computer-assisted sperm analyzer over 24 h. Additionally, the interaction with the spermatozoa was further investigated by means of light and transmission electron microscopy (TEM).

**Results:**

Only viable *Chlamydiae* of both species decreased sperm motility and this only after about 9 h. Taking binding rates into account, the loss of sperm motility after about 9 h could likely be a consequence of *Chlamydiae* attachment to the spermatozoa. About two thirds of the *Chlamydiae* elementary bodies were bound to the front third of the sperm, the acrosomal region. No inclusions of *Chlamydiae* in spermatozoa were observed in TEM after 2 h co-incubation.

**Conclusions:**

As initial motility was not affected following co-incubation of viable *Chlamydiae* and bovine sperm, it seems likely that sperm could serve as a carrier/vehicle for *Chlamydiae* facilitating cervical passage of *Chlamydiae* spp. in cattle. Additionally, our results suggest that spermatozoa carrying *Chlamydiae* may have no initial disadvantage in reaching the oviduct, but are immotile at the time of ovulation what might have an impact on fertilization capacities of the individual sperm. Consequently, high concentrations of the investigated *Chlamydiae* in the seminal plasma or female genital tract might play a role in reduced fertility in cattle.

## Background

*Chlamydia* (*C.*) *trachomatis* infections are known to have a serious impact on the fertility of women, mainly by causing salpingitis [1–3]. In animals, *Chlamydiae* infections may remain asymptomatic, e.g. in cattle [4] or pigs [5], but they were also linked to several reproductive pathologies e. g. vaginitis and endometritis [5–7], mastitis and agalactia [5, 8], salpingitis [9], reproductive failure [5, 10] and abortion [11, 12].

Little is known about the role of *Chlamydiae* spp. in male. The pathogen was detected in semen of several species, like man, bull, ram and boar [13–18]. Infections may cause urethritis and prostatitis [19, 20] but the influence of *Chlamydiae* on male fertility is still controversial. Co-incubation with *C. trachomatis* or chlamydial LPS was shown to cause sperm death [21–23], likely due to increased apoptosis [23]. Some studies have demonstrated *Chlamydiae* infections to be correlated with reduced motility, reduced velocity and increased abnormal morphology of spermatozoa [24–26]. In other studies, however, no significant impact on semen quality and fertility was found in man [27, 28], rat [29], boar [14, 18] and bull [13, 17].

Venereal infection is the classical route for the transmission of *C. trachomatis* in humans [30–33]. There is limited evidence that venereal transmission of *C. abortus* is possible in sheep [15]. Recently, Schautteet et al. [16] reported severe reproductive failure in sows probably related to insemination of *C. suis* contaminated semen. Hamonic et al. [34] confirmed that *C. suis* remains viable and infectious during chilled storage and is more or less unaffected by antibiotics in extenders. The authors consequently hypothesized that extended semen may act as a viable transmission mechanism for *C. suis* in swine [33, 35]. Although it seems obvious that sperm can serve as a vector (vehicle) for *Chlamydiae* to infect the female genital tract, information on interactions between sperm cells and these bacteria is still patchy. There are two studies indicating that, after natural infection of man, *C. trachomatis* penetrates the sperm, preferentially their heads, and can also proliferate within the spermatozoa as indicated by the presence of reticulate bodies [33, 35]; however, the interaction for other host-*Chlamydiae spp.* has not been investigated yet.

To gain further insights into the role of *Chlamydiae* spp. in the bovine, we studied the interaction of *C. abortus* and *psittaci*, *Chlamydiae* spp. previously identified in semen samples of breeding bulls [13, 17], with bovine spermatozoa by means of light and transmission electron microscopy. Furthermore, we monitored the motility of spermatozoa in inoculated semen samples as a parameter directly correlated to fertility.

## Methods

### Animal and semen samples

Semen samples were obtained from an adult, sexually mature black Holstein Friesian bull housed in the Clinic for Obstetrics, Gynecology and Andrology of Large and Small Animals with Veterinary Ambulance in Giessen, Germany (50°35’N 8°40’O). The bull was kept in a 22 m^2^ freestall barn under natural light conditions and temperatures ranging between 15 and 25 °C. He had ad libitum access to water, hay and straw and was additionally fed a commercial diet once a day according to the manufacturer’s instructions. Semen was collected using an artificial vagina (Minitube, Tiefenbach, Germany), and a cow or a bull as dummy. Immediately after collection, the semen samples were examined for sperm concentration as well as for total and progressive motility. For further experiments, samples were diluted to a concentration of 100 × 10^6^ sperm/ml using 35 °C pre-warmed Biladyl® extender without antibiotics (Minitube).

### Chlamydia stock solution

*Chlamydiae* were propagated on Buffalo-Green-Monkey-Kidney cells (ZBV Friedrich-Löffler-Institute, 17,493 Greifswald, Insel Riems, Germany), cultured in sterile filtrated medium [440 ml Eagle’s Minimum Essential Medium supplemented with 0.425 g NaHCO_3_, 5 ml 200 mM L-glutamine (Biochrom GmbH, Berlin, Germany), 5 ml Vitamin 100x (Biochrom GmbH) and 50 ml heat-inactivated fetal bovine serum (FBS, all Biochrom GmbH)] at 37 °C for about 4–7 days. After adding *C. abortus* or *C. psittaci* to the cells, vials were centrifuged for 1 h at 1935 rcf (centrifuge J2–21, rotor JS 7.5, Beckman Coulter GmbH, München, Germany) to promote infection of cells. *Chlamydiae* were allowed to grow for about 4–7 days at 37 °C before being harvested and separated from cell debris as described elsewhere [36]. Analysis with a transmission electron microscope (Zeiss EM 109, Oberkochen, Germany) revealed that the harvested pellet was mainly composed of elementary bodies. *Chlamydiae* concentrations were assessed by counting particles in Gimenez stained smears using an Ortholux II microscope with a counting tube (Leitz Wetzlar, Germany). Briefly, suspensions were diluted 1:50, 1:100 and 1:200 with sterile saline. 10 μl of each dilution were air-dried on a 1 cm^2^ area on a slide and fixated with 100% methanol (Sigma Aldrich, Seelze, Germany) for 1 h. Slides were incubated for 6 min in 0.5 ml carbol fuchsine solution (1.5 mg/ml Neofuchsine, Merck, Darmstadt, Germany, 3 mg/ml phenol, Merck, in phosphate buffer). Afterwards they were rinsed twice with water and counterstained with 0.5 ml malachite green solution (8 g malachite green, Merck, in 1000 ml distilled water) for a minute. Concentration of chlamydial particles was calculated based on the number of particles counted in 100 fields of a counting ocular considering optical magnification (787.5 fold).

### Influence of *Chlamydiae* spp. on total and progressive motility

Ten [10] μl of either a suspension of viable *Chlamydiae* spp. (*C. abortus* or *C. psittaci*, 100 × 10^8^ particles/ml; viability was confirmed by infection of Buffalo-Green-Monkey-Kidney cells), a corresponding suspension of heat-inactivated (95 °C, 1 h) *Chlamydiae* spp., or sterile saline were added to 100 × 10^6^ spermatozoa diluted in 990 μl Biladyl®. The Chlamydia: sperm ratio corresponds to a multiplicity of infection (MOI) of 1. Samples were incubated at 35 °C for 24 h. Every three hours, 3 μl were then transferred into a 20-μl sample chamber (Leja® Standard Count 4 Chamber Slide, 20 μm, Leja Products B. V., Nieuw Vennep, Netherlands) for motility analysis at 37 °C. Percentages of motile and progressively motile spermatozoa were assessed as six repeated measurements with the computer-assisted sperm analyzer (CASA; SpermVisionTM Software Version 3.5.6.2; Minitube) using the settings as given in Table 1. All experiments were performed five times with semen from different preparations.

**Table 1:** Technical settings of the CASA system SpermVision™ for motility analysis

Parameter	Setting
Field-of-view depth = Depth of sample chamber	20 μm
Light adjustment	90–105
Total number of cells evaluated or number of fields	4000 spermatozoa or 8 fields
Sperm recognition area	22–99 μm^2^
Frame rate	60 frames/sec.
Points assessed for sperm motility	11
Total motility	progressive motility + local motility
Immotile sperm	AOC < 5°
Local motility	DSL < 6.0 μm
Progressive motility	Every cell that is not “immotile” or “local motile”
Hyperactive sperm	VCL > 80 μm/s, ALH > 6.5 μm and LIN < 0.65
Linear sperm	STR > 0.5 and LIN > 0.35
Non-linear sperm	STR ≤ 0.5 and LIN ≤ 0.35
Curvilinear sperm	DAP/Radius ≥ 3 and LIN < 0.5

### Chlamydia-sperm interactions

To investigate the interactions of *Chlamydiae* spp. and bovine spermatozoa by light microscopy, 1 ml Biladyl® diluted spermatozoa- *Chlamydiae* suspension (100 × 10^6^ sperm and respective number of *Chlamydiae* spp./ml, *C. abortus* or *C. psittaci*) were incubated at 35 °C for 24 h (long-term co-incubation). In 3-h intervals, 25 μl of the initial suspension were diluted with sterile saline up to 1 ml and centrifuged (tabletop centrifuge, 3 min, 4025 rcf). The supernatant containing unbound *Chlamydiae* was discarded. The pellet was re-suspended with sterile saline and centrifuged again. This centrifugation-washing procedure was performed five times in total. After the last centrifugation step (1935 rcf, 10 min, centrifuge JS-21, Rotor JS-7.5, Beckman Coulter GmbH), spermatozoa were transferred on a cover slide in a 6 ml polystyrene vial (Greiner Bio-One). Afterwards, slides were fixed with 1 ml 100% methanol (Sigma Aldrich) and Gimenez stained (see above). Using a Leica DMRIIC microscope (Leica, Wetzlar, Germany), 50 spermatozoa were examined at a magnification of × 1000. Presence (yes/no) and localization (acrosomal region/remaining other parts of the sperm) of *Chlamydiae* were recorded. The same experiment was performed with samples taken every 30 min over a total of 3 h (short-term co-incubation). All experiments were repeated four times.

For both *Chlamydiae* spp., the presence, localization, and size of chlamydial particles as well as signs of invasion into spermatozoa were further analyzed by electron microscopy. One ml of the above described *Chlamydiae*-sperm suspension (100 × 10^6^ Biladyl® diluted spermatozoa and *C. abortus* or *C. psittaci*/ml) was incubated at 35 °C for 2 h. Removal of unbound *Chlamydiae* was performed as described above. For electron microscopic investigations, the resulting pellets (*n* = 5) were fixed for 12 h with 0.1 mol/l sodium cacodylate buffer (Merck) containing 6% glutaraldehyde (Plano, Wetzlar, Germany). Afterwards, the samples were centrifuged for 10 min at 447 rcf (tabletop centrifuge). The pellets were washed three times with 0.1 mol/l sodium cacodylate buffer. Briefly, they were fixed for 1 h with 1% osmium tetroxide (Plano), dehydrated with ethanol (Merck), embedded in epoxy resin (Plano), sectioned, stained with 0.2% lead citrate and 0.5% uranyl acetate using an Ultrastainer (Leica Reichert, Bensheim, Germany) and evaluated with a transmission electron microscope (Zeiss EM 109, Zeiss, Oberkochen, Germany) at 80 kV. Pure Chlamydia particles in Buffalo-Green-Monkey-Kidney cell culture treated in the same way as indicated above served as positive control.

### Statistical analysis

For all parameters assessed, the different *Chlamydiae* spp., *C. abortus* and *C. psittaci*, were evaluated individually. In general, data was presented as mean ± standard deviation (SD). For statistical analysis, samples were evaluated as repeated measures over the different time points.

To test for a significant effect of *Chlamydiae* spp. on semen motility and progressive motility, respectively, a mixed-effect model for a four-factorial analysis of variance with repeated measures and equal cell sizes was used (fixed factors: treatment and time as repeated measures; random effects: ejaculate and replication; program BMDP8V). A two-way analysis of variance (factors: time and localization) with repeated measures (program BMDP2V) was performed to analyze binding sites of *Chlamydiae* on the sperm, i.e. at the acrosomal region of the sperm head versus any other region of the sperm cell, such as midpiece and tail. Calculations for short- and long-term co-incubation, 3 and 24 h, respectively, were performed separately, excluding the time point 0 (The time when the first sample was taken – time 0).

For all tests, the statistical software program package BMDP Release 8.1 was used [37]. Values were considered to be statistically significant at *P* ≤ 0.05.

## Results

### Influence of chlamydia on total and progressive motility

**Fig. 1:**
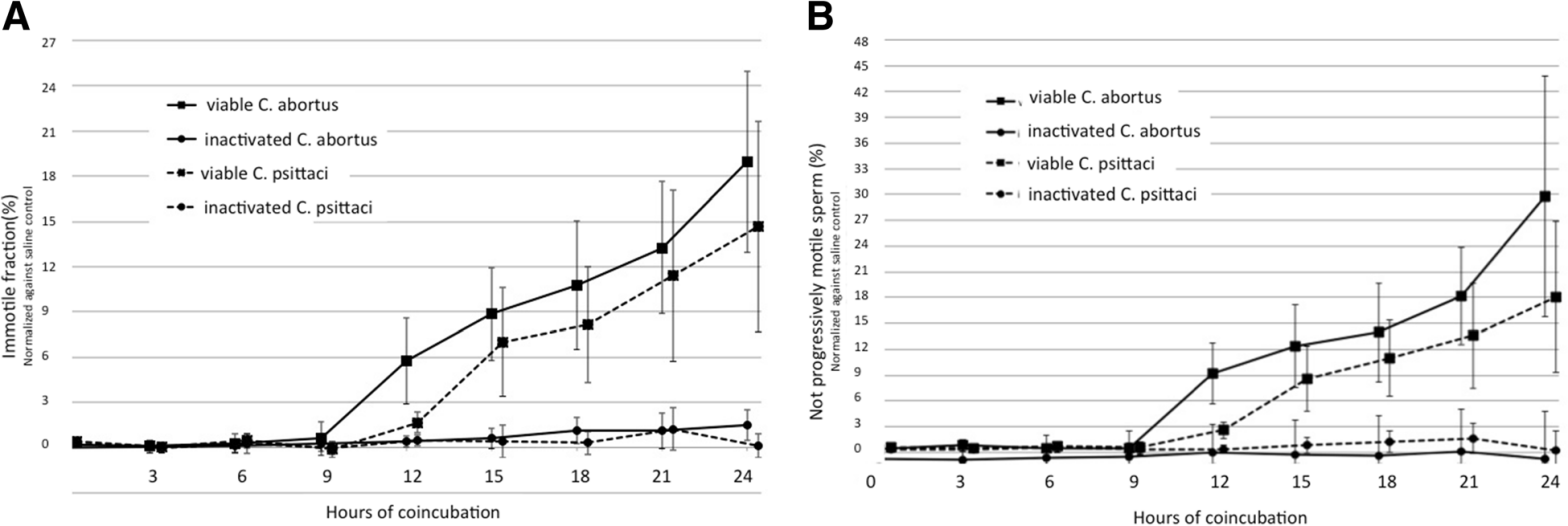
Viable *Chlamydia abortus* und *psittaci* signicantly decrease **a**. total and **b**. progressive motility of bovine spermatozoa. Percentage of immotile bovine sperm (normalized against saline control) coincubated at 35 °C with viable and heat-inactivated Chlamydia (MOI = 1) for 24 h. Results are depicted as mean values ± SD of 5 replicates. Motility of spermatozoa co-incubated with viable *Chlamydiae* spp. was significantly reduced from 9 h onwards compared to motility of spermatozoa incubated with heat inactivated *Chlamydiae* (*p* < 0.0001)

Sperm motility constantly decreased during the 24 h of observation in all experiments. The effect varied strongly between the different ejaculates. Saline controls showed a final reduction of total and progressive motility of 26–90% and 19–81% respectively (data not shown). Nevertheless, test sets from single ejaculates showed a high repeatability. The relative standard deviation within 36 duplicates (saline control, 4 different ejaculates, and 9 different time points) was 0.83%, only. For evaluation of the chlamydial influence on sperm motility, the results for total and progressive motility of the *Chlamydiae-*inoculated samples were related to the results of the respective saline controls (normalized) and presented as the % difference (Fig. 1). Heat inactivated *Chlamydiae* of both species did not significantly reduce sperm motility during the course of the experiment. Spermatozoa co-incubated with viable *Chlamydiae* spp. showed motility results comparable to saline controls in the first nine hours. Interestingly, at all later examination times the immotile fraction was increasingly higher than in the samples inoculated with heat inactivated *Chlamydiae* spp. This effect was statistically significant for both *Chlamydiae* spp. with *P* < 0.0001 after 24 h. The average reduction of the motile fraction was more distinct in samples inoculated with *C. abortus* (19%) than with *C. psittaci* (15%). This effect was even stronger in terms of the mean reduction of progressive motility (*C. abortus* 34%, and *C. psittaci* 18%).

### Chlamydia sperm interaction

**Fig. 2:**
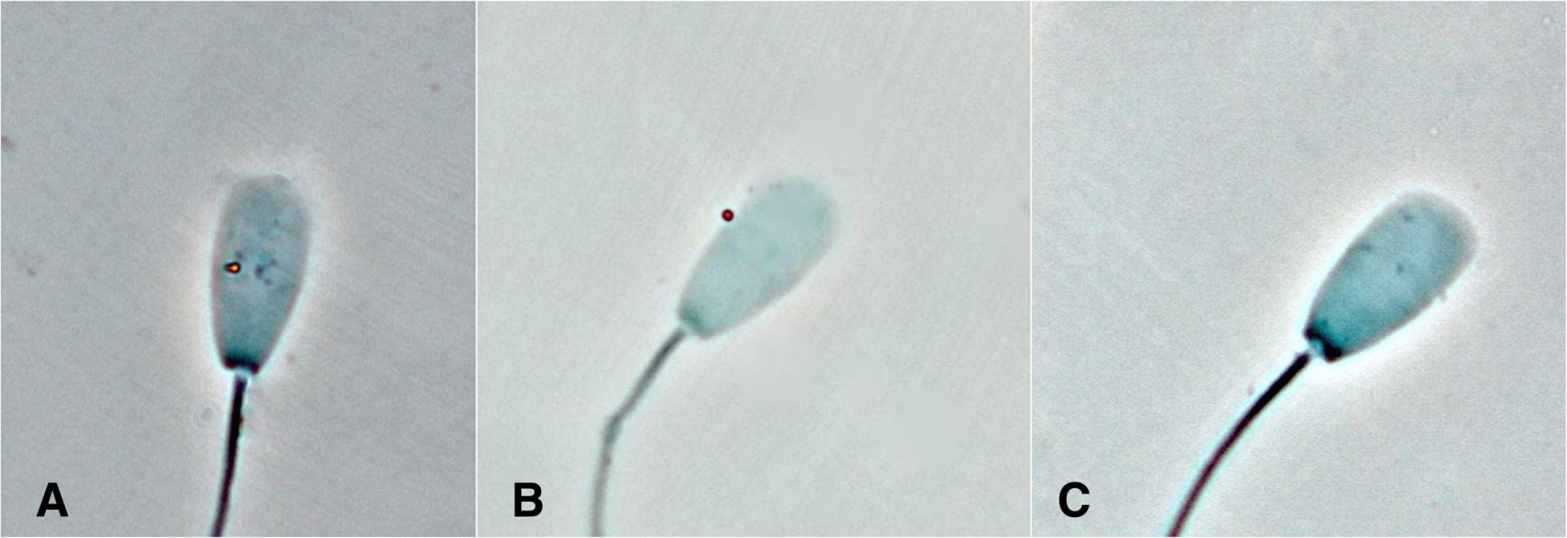
Light microscopical evaluation clearly confirms binding of *C. abortus* and *psittaci* to bovine spermatozoa. The orange particles represent chlamydial particles (**a**. *C. abortus*; **b**. *C. psittaci*) bound to a bovine spermatozoon. The particles look orange due to the use of a blue filter. In the negative control (**c**.), no similar particles are visible (magnification: × 1000)

**Fig. 3:**
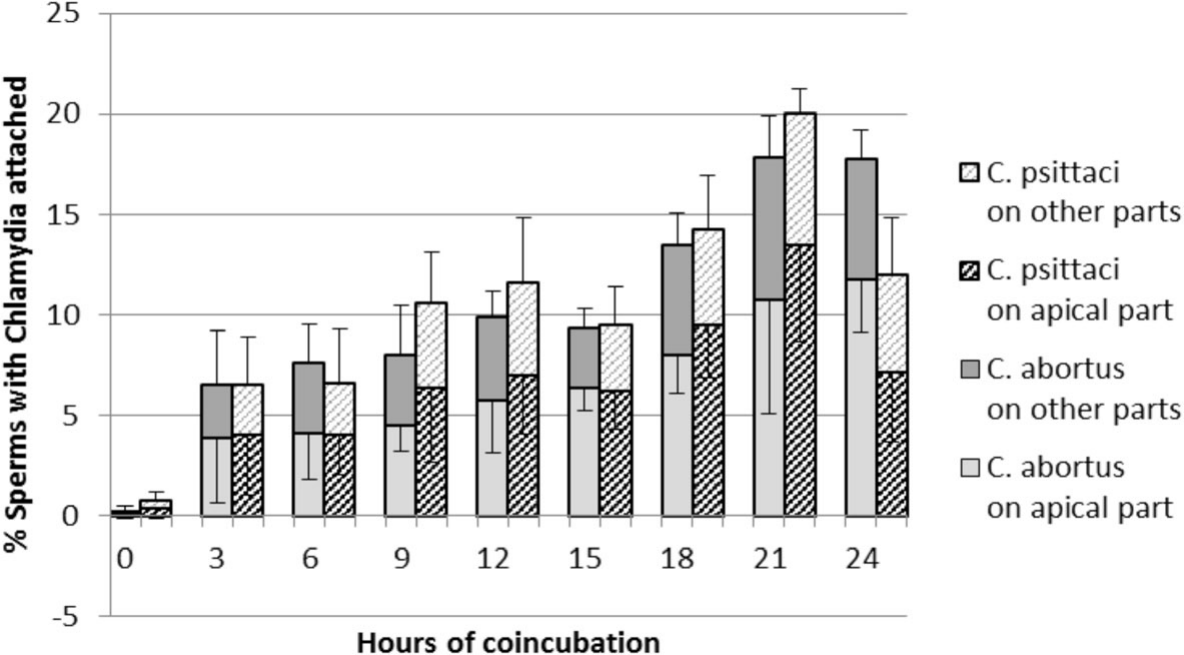
*C. abortus* and *psittaci* can attach to bovine spermatozoa. The apical area of the sperm head seems to be the preferred location for attachment. Attachment of *Chlamydia* spp. (*C. abortus, C. psittaci*,) to the apical (acrosomal) area and other parts of bovine spermatozoa during 24 h of coincubation (MOI = 1) at 35 °C. Results are depicted as mean values of 4 replicates of 50 sperms ± SD. [The top error bar indicates the error bar of “the other parts” for the respective Chlamydia spp. (only positive SD presented); the lower error bar indicates the error bar of “the apical part” (only negative SD presented).] Approximately 60% of *Chlamydiae* spp. particles are attached to the apical area corresponding to the acrosomal region

Light microscopy revealed that viable *C. abortus* and *psittaci* were both able to attach to bovine spermatozoa (Fig. 2) with no significant differences between the two investigated *Chlamydiae* spp*..* Detailed results on the time course and localization of attachment of the two different *Chlamydiae* spp. studied are given in Fig. 3. Binding rates increased in a time-dependent manner. About two thirds of the *Chlamydiae* (in mean 60.3% of *C. abortus* and 62.9% of *C. psittaci*) were found to be attached to the acrosomal region of the sperm head comprising only one third of the sperm surface. In the course of the experiment, this ratio did not change significantly.

**Fig. 4:**
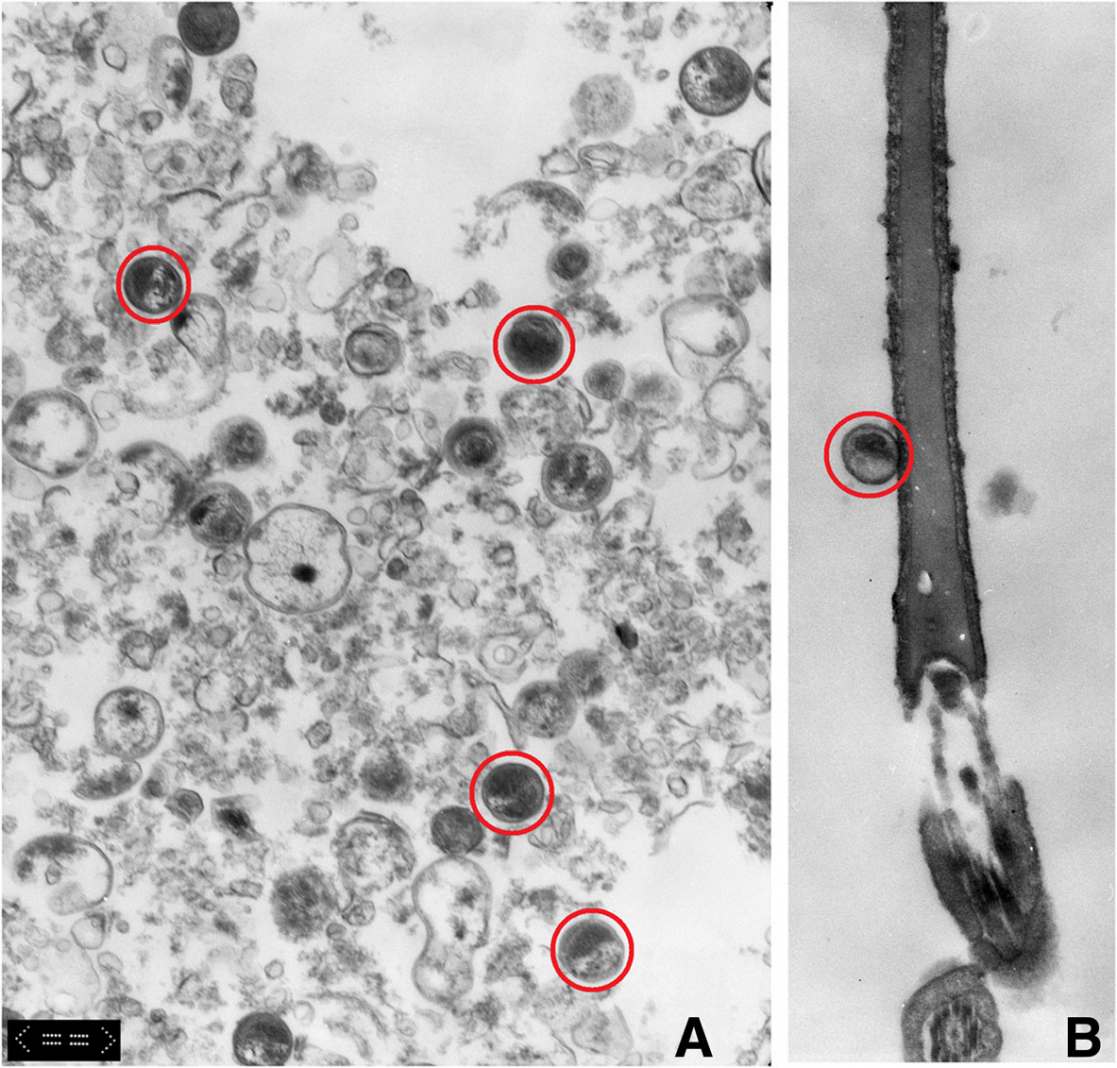
Transmission electron microscopic images showing *Chlamydia*-sperm interaction Exemplary chlamydial particles are marked. **a**. Chlamydial particles in Buffalo-Green-Monkey-Kidney cell culture after harvesting, size indicating elementary bodies, additionally cell debris visible and **b**. a *Chlamydia* spp. particle, resembling an elementary body localised at the apical part of the sperm head (all magnifications M12000, bar indicates 0.6 μm)

Using transmission electron microscopy, *Chlamydiae* were visualized as small round particles of 0.3 to 0.4 μm attached to the spermatozoon’s surface (Fig. 4B). Double membranes surrounded the dark and electron dense particles allowing for easy differentiation from larger cytoplasmic droplets. Chlamydia particles in Buffalo-Green-Monkey-Kidney cell culture pellet were shown after harvesting as positive control (Fig. 4A). Similar to what has been observed with light microscopy (see Fig. 2), most of the *Chlamydiae* seemed to be located at the apical part of the sperm in the acrosomal area. No particles or structures strongly ressembling *Chlamydiae* could be identified inside the sperm head or its nucleus after 2 h of co-incubation by using TEM, nor were any changes in the acrosomal membrane of spermatozoon obvious at the attachment site following 2 h co-incubation.

## Discussion

The present data shows that viable *Chlamydiae* of both investigated species, *C. abortus* and *C. psittaci,* at an MOI of 1 are capable to affect motility of bovine sperm. The effect on motility, a reduction by 15–19%, as observed in this study, is rather impressive, as spermatozoa were inoculated with *Chlamydiae* spp. at an MOI of only 1 resulting in low attachment rates (Fig. 2). Data on natural chlamydial load in semen is rare and, to the best of our knowledge, is not available for bovine ejaculates. Low chlamydial loads, i.e. 1.5 × 10^4^ [38] or 4.9 × 10^4^ [39] particles, were detected in ejaculates of infected men by Real time-quantitative polymerase chain reaction (RT-qPCR). Due to the lack of details about sperm concentration in the aforementioned studies [38–40], it is impossible to compare those with this study in terms of chlamydial load necessary to elicit effects on sperm. However, comparison with the WHO reported minimum and average sperm concentrations in the human ejaculate (9-73 × 10^6^/mL; [41]) reveal possible MOIs between 2.1 × 10^− 4^ and 5.4 × 10^− 3^. Different to the likely low chlamydial load in semen, a higher chlamydial load might be expected in the female genital tract where up to 8.5 × 10^7^ *C. trachomatis* per ml genital fluid had been found in women [39] indicating that similar chlamydial and sperm concentrations as used here, might be possible naturally.

Sperm motility started to decrease nine hours of co-incubation with the respective *Chlamydiae* spp.. In contrast binding of chlamydial particles to sperm was already microscopically observed immediately after the start of co-incubation, and was correlated with time. It seems possible that the negative effect of *Chlamydiae spp.* on total and progressive motility might be due to chlamydial viability, since this effect was absent when spermatozoa were co-incubated with saline only (negative control) or heat-inactivated *Chlamydiae*. Heat-inactivated *Chlamydiae* spp. showed a certain lightmicroscopical integrity and typical Gimenez-staining, however, detailed examinations on the morphology by transmission electron microscopy were not performed as well as details on binding partners/receptors were lacking. These analyses should be included in future studies to identify the binding mechanism. In contrast to this study, other investigations did not find such negative effects on semen parameters, which might be, at least in part, due to differences in the study designs. In particular, semen parameters were evaluated shortly after starting co-incubation (e.g. [27, 28, 35]) at a time where also in this study, no effects of *Chlamydiae spp.* on motility were observed.

Taken together, the results of the current study suggest that spermatozoa start to display a reduced motility around 9 h post co-incubation due to effects of *Chlamydiae* spp.. Moreover, *C. abortus* induced a slightly stronger effect than *C. psittaci*. The reasons for this difference remain to be investigated.

Basic knowledge of the sperms’ fate in the bovine female genital tract is necessary for understanding possible clinical effects of *Chlamydiae*-mediated reduced sperm motility on female reproduction*.* Following male infection, *Chlamydiae* spp. are located in the accessory sex glands in man [19, 20], bulls and boars [42]. During ejaculation, spermatozoa get in contact to the pathogen that is released from the accessory sex glands together with the seminal plasma. In bovines, the spermatozoa are deposited into the anterior vagina during breeding, and are then required to rapidly enter the cervix. However, the cervical passage of sperm is hampered by the cervical mucus, which acts as a mechanical barrier particularly to sperm with abnormal motility patterns or reduced motility thus being a mechanism of sperm selection [43–46]. Additionally, the cervical mucus is also considered to have a filter function for seminal plasma and free microbes [47]. It might thus be that binding to motile spermatozoa is necessary for *Chlamydiae* spp. to not be caught by the cervical mucus in order to be able to reach the uterus and eventually also the oviduct. After having passed the cervix, spermatozoa quickly reach the utero-tubal junction where the apical surface of the sperm head binds to the oviductal epithelium [48, 49] at the site of sperm reservoir (for review see [47, 50]). At this site, sperm remain viable for 18 to 24 h or even longer as earlier studies have shown that artificial insemination with frozen semen (as common in cattle) is most successful, if it is performed in oestrus 12–24 h before ovulation [51]. As motility was first significantly affected after nine hours of co-incubation and the percentages of spermatozoa with bound spermatozoa are similar, it seems, however, likely that *Chlamydiae*–carrying spermatozoa are immotile at the time of fertilization. It remains to be investigated why motility was affected from nine hours after co-incubation and what were the reasons of immotility of spermatozoa. It is noteworthy that heat inactivated *Chlamydiae* spp. had no impact on sperm motility and it deserves further investigation if addition of specific antibiotics to commercial semen extenders or semen freezing is capable to induce the same effects on *Chlamydiae sp..* in semen samples. The inactivation process might have resulted in denaturation of chlamydial structures relevant e. g. for the binding to the host cell, the observed reduction of motility occurred when *Chlamydiae* spp. in other host cells might start intracellular replication, energy parasitism and induction of other severe metabolic changes as a consequence of invasion [4, 52].

Our data did, however, not show an invasion of the studied *Chlamydiae* spp. into the bovine spermatozoa, only adhesion of 0.3–0.4 μm particles considered as infectious elementary bodies was visualised by means of light and transmission electron microscopy. Adherence of chlamydial particles to the sperm surface has been described before for *C. trachomatis* following in vitro [53] and in vivo infection [33, 35], with the latter authors also describing elementary and reticulate bodies within the sperm head [33, 35] and tail [33] by means of transmission and scanning electron microscopy. Interestingly, the described changes resemble previously described genetic or fixative-related membrane changes and defects, crater-like changes in the acrosome and chromatin defects in the sperm head (for review see [54]). It remains to be clarified if the lack of invasion into spermatozoa is real in this *Chlamydiae* spp. – sperm interaction or could have been related to the use of different *Chlamydiae* spp. (*C. trachomatis* versus *C. abortus* and *C. psittaci*) and hosts (human versus bovine), the duration of *Chlamydiae*-sperm interaction (2 h) or due to the fact that the experiments were based on in vitro co-incubation and not on natural infection.

Considering the attachment of *Chlamydiae* spp. to the sperm cells, seminal plasma proteins coating the spermatozoa deserve further attention as binding mediators. As about two thirds of the *Chlamydiae* particles were attached to the apical part of the sperm head, PDC-109 and osteopontin can be considered as strong candidates. Whereas PDC-109 modulates binding to the oviductal epithelium [55–57] and is bound predominantly to the midpiece, but also to the acrosome as well as the post-equatorial and equatorial segments [57], osteopontin, predominantly identified on the acrosome, is hypothesized to be involved in sperm–oocyte interaction, thereby affecting fertilization [57].

## Conclusions

Data shows that viable *C. abortus* and *psittaci* (MOI = 1) attach to spermatozoa. Initially, spermatozoa with attached *Chlamydiae* are not hampered. However, taking binding rates into account, our data also suggests that *Chlamydiae* spp. reduce sperm motility after 9 h of co-incubation in vitro, and thus possibly lead to a reduced fecundity of bull semen. As about two thirds of the *Chlamydiae* particles were bound to the front third of the sperm, the acrosomal region, it is suggested that specific binding proteins, like e.g. PDC-109, are involved in *Chlamydiae*-spermatozoa interaction.
